# Metabolomics as a Tool to Investigate HIV/TB Co-Infection

**DOI:** 10.3389/fmolb.2021.692823

**Published:** 2021-10-20

**Authors:** Chandré Liebenberg, Laneke Luies, Aurelia A. Williams

**Affiliations:** Human Metabolomics, North-West University, Potchefstroom, South Africa

**Keywords:** HIV/AIDS, tuberculosis, HIV/TB co-infection, metabolomics, metabolism, treatment

## Abstract

The HIV/AIDS (human immunodeficiency virus/acquired immunodeficiency syndrome) and tuberculosis (TB) pandemics are perpetuated by a significant global burden of HIV/TB co-infection. The synergy between HIV and *Mycobacterium tuberculosis* (*Mtb*) during co-infection of a host is well established. While this synergy is known to be driven by immunological deterioration, the metabolic mechanisms thereof remain poorly understood. Metabolomics has been applied to study various aspects of HIV and *Mtb* infection separately, yielding insights into infection- and treatment-induced metabolic adaptations experienced by the host. Despite the contributions that metabolomics has made to the field, this approach has not yet been systematically applied to characterize the HIV/TB co-infected state. Considering that limited HIV/TB co-infection metabolomics studies have been published to date, this review briefly summarizes what is known regarding the HIV/TB co-infection synergism from a conventional and metabolomics perspective. It then explores metabolomics as a tool for the improved characterization of HIV/TB co-infection in the context of previously published human-related HIV infection and TB investigations, respectively as well as for addressing the gaps in existing knowledge based on the similarities and deviating trends reported in these HIV infection and TB studies.

## Introduction

Tuberculosis (TB) ranks as the top cause of death due to a single infectious pathogen, ranking above the human immunodeficiency virus (HIV), the causative agent of acquired immunodeficiency syndrome (AIDS) (World Health Organization, 2019). These diseases are pandemics with high morbidity and mortality rates (World Health Organization, 2020). Although HIV-positive individuals suffer from many serious secondary infections, TB is the most common disease, especially in sub-Saharan Africa. Due to the immunosuppressive effects of HIV on the host, HIV-positive individuals have a 26–31 times greater risk of developing active TB compared to HIV-negative individuals (Wood et al., 2000; World Health Organization, 2012). This increased risk, along with various other factors, contributes to the significant global burden of HIV/TB co-infection. Although pulmonary TB is the most common form of active TB in HIV/TB co-infected individuals, this population is also more prone to the development of independent or concurrent extrapulmonary TB, especially when CD4 T cell counts are low (Chaisson et al., 1987; Elliott et al., 1995; Schutz et al., 2010). When co-infection with these pathogens is established, they act synergistically to increase the disease burden in the host (Kwan and Ernst, 2011). As expected, the pathophysiology of *Mycobacterium tuberculosis* (*Mtb*) infection differs in HIV-positive individuals and even more so in those who have progressed to severe immunosuppression, i.e., AIDS, typically showing an accelerated clinical course (Badri et al., 2001; Schutz et al., 2010). In general, HIV/TB co-infected patients present with characteristic symptoms of active pulmonary TB (Burman and Jones, 2003; Luies and du Preez, 2020) but often have more severe systemic symptoms including fevers and weight loss. A mucus-producing cough is less common, which is linked to HIV-induced immunosuppression, as a less vigorous immune response results in fewer pulmonary complications (Selwyn et al., 1998; Garcia et al., 2007; Schutz et al., 2010). In 2019, 208,000 of the approximately 1.4 million TB-related deaths were among HIV-positive people (World Health Organization, 2020).

The incidence of smear-negative (culture-negative, symptomatic) and subclinical (culture-positive, asymptomatic active) TB is also higher among co-infected patients. This leads to decreased sensitivity in screening tests and subsequent diagnostic delays, as the most widely applied TB diagnostic tests are based on sputum examination (Schutz et al., 2010; Tiberi et al., 2017). This manifests as higher mortality rates for smear-negative HIV-positive TB patients, the discovery of subclinical TB only after the initiation of antiretroviral therapy (ART) (Sterling et al., 2010), and poor treatment outcomes for these patients. Therefore, it is recommended that all HIV-positive individuals are screened for *Mtb* infection, latent or otherwise (Tiberi et al., 2017). TB can be cured in these individuals, although treatment is complicated by various factors (Tiberi et al., 2017). Co-infected individuals also have an increased rate of recurrence, mainly due to reinfection as opposed to relapse (Sonnenberg et al., 2001; Charalambous et al., 2008).

Various aspects of the pathology of HIV infection, TB, and HIV/TB co-infection remain incompletely understood, which hampers the development of improved or novel diagnostic and management strategies. There is a need to better understand and characterize the complex metabolic changes induced by co-infection since many deaths can be prevented with timely diagnosis and treatment. Metabolomics is a promising tool in this regard and refers to the analysis of small molecules (i.e. metabolites) present in a biological system, to infer biological functions or changes in response to specific internal or external stimuli (Dunn et al., 2005).

Metabolomics has yielded new insights into HIV and *Mtb* infection- and treatment-induced metabolic alterations experienced by the host. Despite the contributions that metabolomics has made to date, this approach has not yet been systematically applied to characterize the HIV/TB co-infected state. Considering this, we briefly review the HIV/TB co-infection synergism from a conventional and metabolomics perspective. We then evaluate the role of metabolomics as a tool to investigate HIV/TB co-infection in the context of previously published human-related HIV infection (specifically relating to HIV-1) and TB investigations, respectively. This will inform the design of future experiments and allow for a better understanding of the contributions that HIV and *Mtb* make toward host metabolic adaptations during co-infection. Due to the complexity of the synergism between HIV and *Mtb* these metabolic changes might be similar, exacerbated, or altered, compared to HIV infection or TB alone.

## Human Immunodeficiency Virus/Tuberculosis Co-infection: What do We Know and What are the Current Gaps?

HIV and *Mtb* display the same outcome in their interactions with the host: effective replication without killing the host for as long as possible, to improve the chances of transmission to a new host and perpetuation of this process. During co-infection, the core of the synergism, which amplifies the disease burden, lies in the deterioration of immunological functions (Pawlowski et al., 2012; Sharan et al., 2020). The overall immune activation is worse in patients with HIV/TB co-infection relative to those with TB only (Vanham et al., 1996). A detailed discussion of the immunology concerned is outside the scope of this review, however, several publications relating to this topic are available (Toossi, 2003; Diedrich and Flynn, 2011; Kwan and Ernst, 2011; Bell and Noursadeghi, 2018; Sharan et al., 2020).

Considering that immunological deterioration has profound effects on the host metabolism (Cheng et al., 2014; Li et al., 2017), the field of immunometabolism has emerged to better characterize these changes. Immunometabolism refers to the convergence of bioenergetic pathways and the specific functions of immune cells (Shi et al., 2016). To explain briefly, during HIV and *Mtb* infections, the collective actions of the innate and adaptive immune responses result in a concentrated effort to eliminate the pathogenic threats (Kwan and Ernst, 2011). A state of chronic inflammation is established (Semba et al., 2010; Aounallah et al., 2016) and the balance between the immune system and metabolism disrupted. This is mainly due to the significant energy demands and biosynthesis required to mount and sustain an immune response (Aounallah et al., 2016). As such, the infected host experiences anorexia via effects on the hypothalamus (Gautron and Laye, 2010), which collectively results in a negative energy balance, and the consequent utilization of stored fat and eventually protein (from muscle tissue) (Powanda and Beisel, 2003) for energy. This often culminates in malnutrition and the condition known as cachexia (Moldawer and Sattler, 1998), an established feature of both HIV infection and TB. Consequently, there is an inability to control infection, resulting in increased mortality (Zachariah et al., 2002; Paton et al., 2006).

To date, metabolic changes during HIV infection, TB, and HIV/TB co-infection have typically been measured by conventional assays [such as resting energy expenditure measurement by indirect calorimetry (Melchior et al., 1991; Schwenk et al., 2003), body composition measurement by dual-energy X-ray absorptiometry (Meininger et al., 2002; Schwenk et al., 2003) and nitrogen flux by the administration of, for example, a glycine isotope (Paton et al., 2003)]. Using such techniques, HIV infection was characterized by alterations in energy, glucose, lipid, and protein metabolism (Salas-Salvado and Garcia-Lorda, 2001), while active TB was characterized by altered protein metabolism (MacAllan et al., 1998), low serum cholesterol (Taylor and Bamgboye, 1979), and glucose intolerance (Oluboyo and Erasmus, 1990). In HIV/TB co-infected patients, protein metabolism was mainly affected. The net protein balance in the fed state was shown to be impaired, compared to a strongly anabolic protein balance in those with HIV infection or TB only (Paton et al., 2003). Serum albumin levels serve as indicators of protein status. HIV/TB co-infected patients were shown to suffer from hypoalbuminemia relative to TB patients (Van Lettow et al., 2003). The distribution and magnitude of body composition changes were not altered in HIV/TB co-infection relative to that of TB patients (Paton and Ng, 2006).

Although informative, conventional techniques allow for only one specific aspect of metabolism to be investigated at a time (Sitole et al., 2013). This complicates the process of forming a holistic, coherent view of infection- and treatment-induced metabolic alterations. Since metabolomics allows for the measurement of multiple metabolites at a time, it has aided in better understanding HIV infection and TB, respectively. In the case of HIV infection, additional changes in other carbohydrates, amino acids (as well as proteins), lipids, and nucleotides (and/or DNA), has been established by untargeted (Munshi et al., 2013; Williams et al., 2014; Sitole et al., 2015; Li et al., 2018; Sitole et al., 2019) and targeted (Riddler et al., 2008; Williams et al., 2011; Scarpellini et al., 2016; Peltenburg et al., 2018; Sitole et al., 2019) metabolomics studies in an untreated, treated, treatment over time, treatment response and/or comorbidity context (Rodriguez-Gallego et al., 2018; Babu et al., 2019; Rosado-Sanchez et al., 2019). Metabolomics has also been extensively applied to various aspects of TB research, i.e. metabolic mechanisms associated with latent *Mtb* infection and conversion to active disease, *Mtb* infection at the cellular level, active TB, comorbidities, as well as TB treatment (Luies et al., 2017; Vrieling et al., 2019; Albors-Vaquer et al., 2020; Vrieling et al., 2020). The approach has thus contributed, among others, to a better understanding of the pathogen, its interaction with the host, and its effect on the physiology of the host. Many studies have focused on the discovery of biomarkers for specific TB-related applications, but the results are often not extensively interpreted in a biological context. Metabolomics, however, has not yet been systematically applied to characterize the co-infected state.

It is worth mentioning that the metabolic characteristics of HIV/TB co-infection derived from conventional methods mainly focused on the synergistic effects of HIV infection, TB, and malnutrition, which together have been termed “triple trouble” with reference to the public health term “double trouble” when referring to HIV/TB co-infection (Van Lettow et al., 2003). The risk of malnutrition (Swaminathan et al., 2008) and the severity of wasting (Lucas et al., 1994; Villamor et al., 2006) are exacerbated during HIV/TB co-infection relative to either disease state alone, although the mechanisms underlying this are not fully understood. HIV infection is associated with the loss of gut mucosal integrity. The CD4 T cells of the gut mucosa are preferentially depleted, leading to impaired immune function in the gut (Ibarrondo et al., 2013; Sharan et al., 2020). This leads to a “leaky gut” and the translocation of gut microbial products into the blood, which exacerbates systemic immune activation (Brenchley et al., 2006; Sharan et al., 2020). Furthermore, it is well established that HIV induces changes in the gut microbiota composition (Vujkovic-Cvijin and Somsouk, 2019). How these processes are affected by *Mtb* during HIV/TB co-infection are not yet understood (Waters et al., 2020), although it is known that patients with TB also have an altered gut microbiota composition (Winglee et al., 2014; Luo et al., 2017; Hu et al., 2019).

The effects of HIV infection on the gut and the high energy requirement during chronic infection culminates in micronutrient deficiencies [reviewed in detail by Semba et al. (2010)] and alterations to the macronutrient status (e.g., increased utilization of protein and fat for energy). Micronutrients have major roles in maintaining homeostasis. Deficiencies have far-reaching complications for the immune system and metabolism (Katona and Katona-Apte, 2008), of which tracing the source might be extremely difficult due to the confounding effect of the inflammatory state and the inherent interconnectedness of metabolism.

Although aspects of metabolism and nutrition improve by treating HIV-positive or TB patients with ART and anti-TB drugs, respectively (Kennedy et al., 1996; Drain et al., 2007), these treatments do not restore patients to a healthy state. HIV-positive individuals often still suffer from wasting even after the initiation of ART (Tang et al., 2002). ART also induces metabolic alterations separate from HIV infection (van der Valk et al., 2002; Jain et al., 2005), while anti-TB treatment fails to completely recover protein reserves (Onwubalili, 1988; Schwenk et al., 2004) and induces oxidative stress (OS) (Sodhi et al., 1997). These effects are reflected in the metabolomics literature for HIV infection (Hewer et al., 2006; Li et al., 2018; Babu et al., 2019) and TB (Luies et al., 2017; Combrink et al., 2019; Opperman et al., 2021). The causes of malnutrition and wasting in HIV-positive and TB patients are multifactorial and are influenced by disease progression. It is known that the development and degree of HIV-induced wasting is influenced by factors such as CD4 T cell count (Forrester et al., 2001), viral load (Lyles et al., 1999; Zackin et al., 1999), inflammatory markers [reviewed by Chang et al. (1998)], and nutrition [reviewed by Koethe and Heimburger. (2010)]. The role of these factors and the HIV-induced gut complications mentioned above, in the co-infected individual is not well understood.

The signaling pathways involved in pathogenic control are closely involved in the regulation of nutrients and metabolic homeostasis (Aounallah et al., 2016) and are mediated by cytokines, chemokines, and various reactive oxygen species (ROS). Besides directly damaging the invading microbe, ROS stimulates the production of pro-inflammatory cytokines, which exacerbates the inflammatory state and perpetuates immunometabolic alterations (Zhang et al., 2018). Increased levels of ROS and pro-inflammatory cytokines are the hallmarks of chronic inflammation (Awodele et al., 2012), and have been studied in HIV infection (Williams et al., 2013), TB (Yao et al., 2017), and co-infection (Verma et al., 2018). Although these interactions have been linked to various metabolic changes and are more pronounced during co-infection (Madebo et al., 2003; Awodele et al., 2012), these are complex and not yet fully understood (Zhang et al., 2018).

No single factor can be attributed to the cause of the co-infected phenotype as the interactions between the host and the two pathogens are multidirectional: either oppositional or synergistic and to various degrees. Given that conventional assays are less sensitive, less specific, and laborious to perform (Williams et al., 2011), researchers have turned to newer, high-throughput omics techniques to better match the dimensionality of the problem to the data obtained and thus allow for a more comprehensive profile of host metabolism, as it relates to HIV/TB co-infection.

## Human Immunodeficiency Virus/Tuberculosis Co-Infection and Metabolomics

Only three metabolomics investigations that study a very specific aspect of HIV/TB co-infection have been published to date (Table 1). To the best of our knowledge, no metabolomics study has hitherto characterized the HIV/TB co-infected metabolic profile relative to healthy individuals or either HIV-positive or TB-positive individuals, or in terms of a treatment-associated profile.

**TABLE 1:** Metabolomics studies published to date investigating a specific aspect of HIV/TB co-infection.

Research Model	Aim	Metabolome fraction targeted	Analytical apparatus	Groups compared	Statistical approaches	References
Human: plasma	To investigate whether IDO activity (measured as Trp/Kyn) could be used as a diagnostic/predictive marker of TB in HIV-positive individuals	Tryptophan and kynurenine	UPLC-MS	*HIV+/TB+/ART+	*t*-test, Mann-Whitney U, Kruskal-Wallis, AUC of ROC, Spearman correlations	Adu-Gyamfi et al. (2017)
				*HIV+/TB+/ART+/ATT+		
				HIV+/TB-/pneumonia+		
Human: plasma	To investigate whether individuals with and without TB-IRIS could be distinguished based on metabolic differences and if these differences are detectable before the onset of TB-IRIS	Total metabolome	LC-MS	HIV+/TB+/ART+/non-IRIS	*t*-test with Bayesian adjustment, FC, PCA, pathway analysis in MetaboAnalyst, Lasso regression models	Silva et al. (2019)
				HIV+/TB+/ART+/IRIS+		
Human: plasma	To determine whether tryptophan metabolism is among the highest regulated metabolic pathways in the response of the host to *Mtb* infection, TB, and treatment	Total metabolome	LC-MS, MS/MS	HIV-/LTBI- (healthy controls)	Wilcoxon rank, AUC of ROC, pathway enrichment analysis	Collins et al. (2020)
				*HIV-/LTBI+/TB-/DOTS+		
				*HIV-/TB+/DOTS+		
				*HIV-/MDR-TB+/MDR-Tn+		
				*HIV+/MDR- TB+/MDR-Tn+		

Aim: IDO: indoleamine 2,3-dioxygenase; Kyn: kynurenine; Trp: tryptophan; TB: tuberculosis; IRIS: immune reconstitution inflammatory syndrome.

Analytical apparatus: (UP)LC-MS: (ultra-performance) liquid chromatography mass spectrometry; MS/MS: tandem mass spectrometry.

Groups: HIV: human immunodeficiency virus; TB: tuberculosis; ART: antiretroviral treatment; ATT: anti-TB treatment; IRIS: immune reconstitution inflammatory syndrome; +: positive; -: negative; *: longitudinal study; LTBI: latent *mycobacterium tuberculosis* infection; DOTS: directly observed treatment, short-course; MDR: multidrug-resistant tuberculosis; MDR-Tn: treatment for MDR-TB.

Statistical approaches: AUC of ROC: area under the curve of the receiver operating characteristic; FC: fold change; PCA: principal component analysis.

The optimal physiological function is reliant on a balanced tryptophan metabolism (Wang et al., 2021), as the immune, central nervous, and reproductive systems respond to alterations in its catabolic flux (Ball et al., 2014). The enzymes tryptophan 2,3-dioxygenase and indoleamine 2,3-dioxygenase 1 (IDO1) catabolize tryptophan via the kynurenine pathway, producing a range of immunomodulatory and neuroactive catabolites. IDO1 activity in particular has been widely investigated for its role in immune tolerance and suppression (Wang et al., 2021). Increased IDO1 activity, induced by interferon-γ (Katz et al., 2008), and the resulting alterations to the kynurenine/tryptophan (K/T) ratio have been investigated as biomarkers for various disease states (Sucher et al., 2010; Mancuso et al., 2015; Pett et al., 2017; Li et al., 2019), including HIV infection and TB measured conventionally (Almeida et al., 2009; Jenabian et al., 2013; Gostner et al., 2015; Dagenais-Lussier et al., 2016; Gautam et al., 2018; van Laarhoven et al., 2018) and via metabolomics (Weiner et al., 2012; Luies and Loots, 2016; Isa et al., 2018; Li et al., 2018; Peltenburg et al., 2018; Sitole et al., 2019).

Of the three studies mentioned, two investigated the effects of IDO1 activity on host metabolism during HIV/TB co-infection. Adu-Gyamfi et al. (2017) monitored HIV-positive individuals longitudinally for the development of active TB. At the time of diagnosis, IDO1 activity was higher in those individuals who developed TB compared to those who did not, an effect that was reversed after 6 months of anti-TB treatment. The IDO1 activity of HIV/TB co-infected individuals was also four-fold higher when compared to that of HIV-positive individuals with confirmed pneumonia. Collins et al. (2020) confirmed that individuals with HIV/TB co-infection have higher IDO1 activity compared to those with a single disease. Collins et al. (2020) further found that HIV-positive individuals with multidrug-resistant (MDR) TB also had an increased K/T ratio relative to those with TB alone. After receiving treatment for MDR-TB for 2 years, there was no significant change in the K/T ratio, which is in concurrence with a slower bacterial clearance rate in MDR-TB patients (Collins et al., 2020). Indoleamine 2,3-dioxygenase 2 (IDO2) plays a smaller role in tryptophan catabolism and has been suggested to promote pro-inflammatory B cell responses, as opposed to the immunosuppressive effects of IDO1, through the induction of T regulatory cell responses (Manches et al., 2008) and other mechanisms (Munn et al., 1999; Mellor et al., 2002; Merlo et al., 2020). The levels of IDO2 transcripts is higher during co-infection compared to individuals with TB alone, suggesting that HIV infection or the co-infected state causes increased production of IDO2 in addition to IDO1 (Collins et al., 2020). These studies indicate that increased IDO activity is significantly more pronounced in the HIV/TB co-infected state and that the K/T ratio may be a suitable biomarker for TB in HIV-positive patients (Adu-Gyamfi et al., 2017) and a possible target for host-directed therapies (Collins et al., 2020). Studies are needed to evaluate whether the significantly increased IDO1 activity contributes to the increased TB progression risk in HIV-positive individuals (Pawlowski et al., 2012; Collins et al., 2020) and those studies comparing HIV-positive individuals with other secondary (specifically respiratory) diseases as diseased controls to validate this are also warranted. Diagnostically, it would be ideal to include both HIV-negative TB-positive patients and HIV-positive TB-negative patients as controls in a single study.

Within the context of co-infection, ART is typically initiated based on a patient’s immunological status. Patients with CD4 <50 cells/mm^3^ or significantly advanced disease are prescribed to start ART within 2 weeks of starting anti-TB treatment, while those with an improved immunological profile (CD4 >50 cells/mm^3^) start ART two to 8 weeks after anti-TB treatment initiation. These guidelines have been proposed in response to the development of an aberrant immune response (immune reconstitution inflammatory syndrome; IRIS) in HIV/TB co-infected patients when ART is initiated early in the course of anti-TB treatment (Török et al., 2011; Manosuthi et al., 2012; World Health Organization, 2016b). IRIS is characterized by a severe inflammatory response that is triggered against a pre-existing condition in HIV-positive individuals shortly after the initiation of ART. When this reaction is directed against *Mtb*, it is referred to as TB-IRIS. It may be transient and resolve of its own, but it may also be fatal. Since there are currently no diagnostic tests for TB-IRIS, its diagnosis is based on clinical presentation and medical history (Pozniak et al., 2011; Tiberi et al., 2017). Considering this, Silva et al. (2019) collected samples from HIV/TB co-infected individuals after the initiation of ART and as close as possible to the onset of IRIS (at a time point compared to this for those who did not develop IRIS). Those individuals who developed IRIS experienced increased arachidonic acid (most notably increased hydroxyeicosatetraenoic acids) and glycerophospholipid metabolism relative to the non-IRIS group at the start of the study, whereas altered sphingolipid and linolenic acid metabolism distinguished the groups during the IRIS window. This study showed that altered polyunsaturated fatty acid metabolism distinguished patients who develop IRIS from those who do not. Since IRIS involves an acute inflammatory response these results are expected, as polyunsaturated fatty acids give rise to various inflammatory mediators. This study again underscores the reflection of immunological changes in the metabolism and its potential prognostic application.

The studies reviewed here stress the many gaps that exist in our knowledge of HIV/TB co-infection, including an incomplete understanding of the mechanisms underlying diagnostic problems, the effects of ART and anti-TB treatment on the host, recurrent TB, and the effect of *Mtb* on the gut mucosal damage induced by HIV. These studies also highlight the potential of metabolomics to address such gaps. Comparing the existing HIV infection and TB metabolomics profiles (Tables 2–4) in the absence of a comprehensive HIV/TB profile may yield insight into the mechanisms of what is known about host metabolism during HIV/TB co-infection and inform the design of future metabolomics studies.

**TABLE 2:** A comparison of the findings of HIV infection and TB serum metabolomics investigations.

Similarities							
Metabolic alteration		Proposed origin(s) of metabolic alterations^a^				References	
Decreased tryptophan and increased levels of its catabolic products (e.g. kynurenine)		Increased IDO activity^b^				HIV: Sitole et al. (2019)	
						TB: Weiner et al. (2012)	
Altered indole-3-acetic acid		Uremic cytotoxic activity, gut microbes				HIV: Williams et al. (2011)	
						TB: Weiner et al. (2018)	
Increased glutamate		*Mtb* nitrogen balance, redox homeostasis, and survival mechanisms. Immune activation and oxidative stress of the host				HIV: Sitole et al. (2019)	
						TB: Zhou et al. (2013); Cho et al. (2020)	
Increased aspartate		Immune activation and oxidative stress				HIV: Sitole et al. (2019)	
						TB: Weiner et al. (2012); Cho et al. (2020)	
Altered phenylalanine and tyrosine		Decreased activity of phenylalanine hydroxylase				HIV: Sitole et al. (2019)	
						TB: Weiner et al. (2012); Che et al. (2013); Zhou et al. (2013)	
Decreased alanine		Energy production				HIV: Sitole et al. (2019)	
						TB: Zhou et al. (2013)	
Altered pyroglutamic acid/5-oxoproline		Mitochondrial dysfunction, glutathione deficiency (i.e. oxidative stress), or reduced glutathione cycling				HIV: Williams et al. (2011)	
						TB: Weiner et al. (2012); Che et al. (2013)	
Altered phospholipids		*Mtb*-induced inhibition of phospholipase A2, lipolysis in the granuloma, inflammation, and immune activation				HIV: Williams et al. (2011)	
						TB: Weiner et al. (2018)	
Increased fatty acids and/or their catabolic products		Inflammation and immune activation, mitochondrial dysfunction				HIV: Williams et al. (2011)	
						TB: Weiner et al. (2018)	
Decreased creatine						HIV: Sitole et al. (2019)	
						TB: Weiner et al. (2018)	
**Differences**							
HIV infection	Proposed origin(s) of metabolic alterations		References	TB	Proposed origin(s) of metabolic alterations		References
				Altered urea cycle intermediates			Weiner et al. (2012); Che et al. (2013)
				Decreased methionine, increased oxidative products of methionine	Oxidative stress		Weiner et al. (2012); Cho et al. (2020)
				Increased fibrinopeptides	Tuberculous granulomas		Weiner et al. (2012)

a Observed trends are not always attributed to a specific mechanism by the investigators. Thus, metabolite changes may overlap between the HIV infection and TB metabolic profiles, but the proposed mechanism given in the table may represent only that given in either the HIV or TB literature.

b IDO: indoleamine 2,3-dioxygenase.

**TABLE 3:** A comparison of the findings of HIV infection and TB plasma metabolomics investigations.

Similarities						
Metabolic alteration			Proposed origin(s) of metabolic alterations^a^			References
Decreased tryptophan and increased levels of its catabolic products (e.g. kynurenine)			Increased IDO activity^b^			HIV: Li et al. (2018); Peltenburg et al. (2018)
						TB: Vrieling et al. (2018); Vrieling et al. (2019); Collins et al. (2020)
Altered glutamine and/or glutamate			Involved in multiple overlapping metabolic pathways			HIV: Munshi et al. (2013); Tarancon-Diez et al. (2019)
						TB: Vrieling et al. (2018); Vrieling et al. (2019); Ding et al. (2020)
Increased aspartate			Dysregulated urea cycle, involved in many other metabolic processes			HIV: Munshi et al. (2013); Scarpellini et al. (2016)
						TB: Vrieling et al. (2019); Ding et al. (2020)
Dysregulation of the urea cycle (decreased ornithine and related metabolites which do not correspond exactly)			Dysregulation of amino acid metabolism by various mechanisms			HIV: Peltenburg et al. (2018)
						TB: Vrieling et al. (2019)
Decreased alanine			Wasting/cachexia			HIV: Peltenburg et al. (2018)
						TB: Vrieling et al. (2018); Vrieling et al. (2019)
Decreased histidine			Oxidative stress and/or inflammation			HIV: Li et al. (2018)
						TB: Vrieling et al. (2018); Vrieling et al. (2019); Ding et al. (2020)
Altered choline						HIV: Munshi et al. (2013)
						TB: Vrieling et al. (2019)
Increased sarcosine			Muscle wasting and folate deficiency during HIV			HIV: Munshi et al. (2013), Peltenburg et al. (2018)
						TB: Vrieling et al. (2019)
**Differences**						
**HIV infection**	**Proposed origin(s) of metabolic alterations**	**References**		**TB**	**Proposed origin(s) of metabolic alterations**	**References**
Altered BCAAs and their catabolic intermediates (↑ during early stages of infection, ↓ during later stages)	Immune activation, muscle catabolism (wasting)	Li et al. (2018); Peltenburg et al. (2018); Tarancon-Diez et al. (2019)		Altered phenylalanine		Vrieling et al. (2018); Vrieling et al. (2019); Ding et al. (2020)

a Observed trends are not always attributed to a specific mechanism by the investigators. Thus, metabolite changes may overlap between the HIV infection and TB metabolic profiles, but the proposed mechanism given in the table may represent only that given in either the HIV or TB literature. BCAA: branched-chain amino acid; ↑: increased; ↓: decreased.

b IDO: indoleamine 2,3-dioxygenase.

**TABLE 4:** A comparison of the findings of HIV infection and TB urinary metabolomics investigations^a^.

Similarities					
Metabolic alteration		Proposed origin(s) of metabolic alterations			References
Increased neopterin		Product of the catabolism of guanosine triphosphate in immune cells in response to interferon-gamma — indicative of a pro-inflammatory immune status			HIV: Munshi et al. (2013)
					TB: Isa et al. (2018)
Increased catecholamines (e.g. epinephrine, norepinephrine)		Various roles in the immune system and as neurotransmitters and hormones			HIV: Munshi et al. (2013)
					TB: Das et al. (2015)
**Differences**					
HIV infection	Proposed origin(s) of metabolic alterations	References	TB	Proposed origin(s) of metabolic alterations	References
Increased isoleucine and methionine, decreased alanine	Immune activation, increased amino acid oxidation for energy	Munshi et al. (2013)*	Increased tryptophan catabolic products (e.g. kynurenine)	Increased IDO activity	Luies and Loots (2016), Isa et al. (2018)
Increased formic acid and decreased 2-methylglutaric acid	Altered central energy metabolism; pyruvate and TCA cycle metabolism		Increased intermediates of phenylalanine and tyrosine metabolism	Compromised insulin production and resulting in decreased PAH activity, altered gut microbial activity	Luies and Loots (2016); Das et al. (2015)
			Increased fatty acids and their catabolic products	Compromised insulin production, various bacterial mechanisms	Luies and Loots (2016)
			*Mtb*-specific metabolites	Cell wall components, intermediates of bacteria-specific metabolic pathways	Luies and Loots (2016)

a There is only one HIV metabolomics study on urine that focused on the characterization of the disease state. IDO: indoleamine 2,3-dioxygenase 1; PAH: phenylalanine hydroxylase; TCA: tricarboxylic acid cycle.

## Comparing Human Immunodeficiency Virus Infection and Tuberculosis Metabolic Profiles

To date, metabolomics has been used to investigate various aspects of HIV infection and TB, using various sample matrices, which can be used as a guide in terms of what to expect during co-infection. HIV infection metabolomics studies have often been concerned with characterizing the disease state, prognosis, and the effect of ART, using blood-based (serum or plasma), cerebrospinal fluid (CSF), cell culture, urine, bronchoalveolar lavage fluid (BALF), oral wash/saliva, and tissue samples (Williams et al., 2017). Within the TB metabolomics literature, blood-based, urine, sputum, tissue, pleural effusion, and cell culture samples have been used, with a focus on biomarker discovery for diagnosis and treatment outcome. As *Mtb* has its own metabolism, unlike HIV, bacterial culture metabolomics has also been extensively used to characterize the *mycobacterium*’s metabolic adaptations (Du Preez et al., 2019).

The normal course of the interactions between the metabolic, immune and endocrine systems is distorted due to the chronic nature of immune activation and inflammation during HIV and *Mtb* infection. At the time of diagnosis, when treatment-naïve samples are typically collected, overlapping biochemical processes have already been set in motion and become so interconnected that no single measure of causality can be attributed. This makes it difficult to interpret metabolomics data. Despite this, and although the trends are not always consistent, an overlapping pattern of metabolites frequently altered during HIV infection and TB can be identified and linked to known effects of these infections. Tables 2–4 compare the serum, plasma and urine metabolomics profiles of untreated HIV-positive and TB patients (where these patients were compared to healthy controls), respectively. Other sample matrices were not compared in this way as there is very little overlap in the use of matrices other than blood and urine between the two disease states. In cases where a metabolite was found to be significantly altered during HIV infection and/or TB, but where the trend was inconsistent, the metabolite is marked as “altered,” instead of “increased” or “decreased.”

Despite the potential for metabolic differences between serum and plasma (Liu et al., 2018), the blood-based metabolomics investigations reviewed here show that there is significant overlap between the metabolic alterations, as induced by HIV infection and TB. For example, alterations to tryptophan and its catabolic products, glutamine and/or glutamate, aspartate, as well as alanine are commonly reported for HIV infection and TB in both serum and plasma (see “similarities” in Tables 2 and 3). Alterations to the following metabolites are similar (being frequently cited as statistically significant and, in some cases, trending in the same direction) between HIV infection and TB but occur only in either serum or plasma: indole-3-acetic acid, phenylalanine and tyrosine, pyroglutamic acid (also called 5-oxoproline), phospholipids, fatty acids, and creatine in serum (Table 2), as well as urea cycle intermediates (other than aspartate), histidine, choline, and sarcosine in plasma (Table 3).

Amino acids are central to metabolism and the function of the immune system, especially during infection. As an infection becomes chronic, these alterations become more complex as various other areas of metabolism become impaired, and amino acids may antagonize each other’s effects (Li et al., 2007). Besides the specific functions of amino acids in the immune system, free amino acids, as well as stored amino acids released from muscle tissue, are used as a last resort to produce energy via the tricarboxylic acid cycle (TCA) cycle during extended periods of high energy expenditure, such as chronic infection. Glutaminolysis has been postulated as a mechanism by which the TCA cycle is replenished during viral infection (Plaza et al., 2016). Continual oxidation of amino acids combined with malnutrition and HIV-associated malabsorption (Grinspoon and Mulligan, 2003) further contributes to alterations in amino acid metabolism. Cachexia is associated with progressive HIV infection and TB and is characterized by the loss of muscle mass. As such, the mobilization of amino acids for energy production may contribute to the observed amino acid changes.

The interconversion of glutamine and glutamate (Tables 2 and 3) is important in metabolic reactions essential to cellular function (Newsholme et al., 2003). Although alterations to both these metabolic partners are not always statistically significant (likely due to the untargeted nature of studies), one or both of these metabolites are a common feature of HIV infection (Munshi et al., 2013; Sitole et al., 2019; Tarancon-Diez et al., 2019) and TB metabolic profiles (Zhou et al., 2013; Vrieling et al., 2018; Vrieling et al., 2019; Cho et al., 2020). Where these partners are found together, increased levels of glutamate with a concurrent decrease in glutamine results in a decreased glutamine/glutamate ratio (Cho et al., 2020; Ding et al., 2020). The alterations to glutamine and glutamate have been attributed to nitrogen balance, redox homeostasis and survival mechanisms of *Mtb* (Frediani et al., 2014; Cho et al., 2020; Thandi et al., 2020). It has also been associated with immune activation, stress on the host, and has been indicated as an early metabolic alteration during HIV (Tarancon-Diez et al., 2019) and *Mtb* (Albors-Vaquer et al., 2020) infection.

The balance of glutamine and glutamate, as well as asparagine and aspartate, have various roles in the immune system. An imbalance in these ratios affects the immune-competence of T cells and macrophages (Yoneda et al., 2009). Increased serum and plasma aspartate was reported for HIV (Munshi et al., 2013; Scarpellini et al., 2016; Sitole et al., 2019). An increased aspartate/asparagine ratio was also observed in the serum (Table 2) and plasma (Table 3) of TB patients (Cho et al., 2020; Ding et al., 2020), confirming the finding of increased aspartate by Weiner et al. (2012).

Aspartate forms part of the urea cycle, where it is combined with citrulline to form argininosuccinate by argininosuccinate synthase. In the liver mitochondria, aspartate is also produced from oxaloacetate by aspartate aminotransferase, with the concurrent conversion of glutamate to α-ketoglutarate (Watford, 2003). This highlights the interconnected nature of the observed alterations to glutamine/glutamate and aspartate in HIV infection and TB metabolomics. Since part of the urea cycle occurs in mitochondria, these alterations relate to mitochondrial dysfunction that has previously been associated with HIV infection (Pinti et al., 2010) and TB (Luies et al., 2017; Ramond et al., 2019). Alterations to the urea cycle were only reported for TB in serum (Table 2) but not for HIV infection. Similar urea cycle alterations were reported for both HIV infection and TB in plasma (Table 3). Decreased urea (Weiner et al., 2012), ornithine (Vrieling et al., 2019), and citrulline, with concurrently increased aspartate (Weiner et al., 2012; Vrieling et al., 2019; Ding et al., 2020) are indicative of urea cycle dysregulation in TB patients. Although the exact mechanisms underlying these alterations are still unclear, they may be related to antimicrobial mechanisms of immune cells. For example, Thandi et al. (2020) showed that ornithine inhibits *Mtb* growth and enhances autophagy in mouse alveolar macrophages. Reduced levels of ornithine and putrescine suggest a decreased urea cycle and reduced ammonia clearance in HIV-positive individuals (Peltenburg et al., 2018).

Alterations to plasma phenylalanine are more commonly reported for TB than for HIV infection [see “similarities” (Table 2) and “differences” (Table 3)], with only one HIV serum investigation reporting on this metabolite (Table 2). These changes have been postulated to be related to decreased activity of phenylalanine hydroxylase, which converts phenylalanine to tyrosine (Ghannoum et al., 2013), as a consequence of compromised insulin secretion (Luies and Loots, 2016). Inflammatory diseases (such as TB) are often associated with insulin resistance, hence diabetes mellitus (DM) is a known risk factor for TB. Evidence for insulin resistance has also been found in TB patients without a clinical diagnosis of DM, indicating that the condition is caused by the diseased state (Philips et al., 2017). Two recent metabolomics studies by Vrieling et al. (2018); Vrieling et al. (2019) characterized the metabolic alterations underlying TB and DM, as well as how these change when DM presents as a comorbidity in TB patients. These studies confirmed previous reports of increased serum (Weiner et al., 2012; Zhou et al., 2013) and urinary (Luies and Loots, 2016) phenylalanine levels in TB patients. One serum-based study (Che et al., 2013), however, reported decreased phenylalanine. Altered energy regulation through the endocrine system may also contribute to the other metabolic alterations discussed here, as various enzymes are influenced by insulin (Dashty, 2013). Furthermore, mitochondrial metabolism, which is modulated by HIV and *Mtb*, is central to coupling amino acids (such as glutamine, glutamate, and alanine) to insulin secretion (Newsholme et al., 2007).

During gluconeogenesis, non-carbohydrate molecules are converted to glucose, an essential energy substrate for leukocytes (Newsholme and Newsholme, 1989) during times of decreased energy availability. In the liver, the glucose-alanine cycle functions as a gluconeogenesis pathway, using alanine as a substrate. The amino group of alanine is transaminated to a keto acid, and the resulting carbon skeleton used in gluconeogenesis. This process contributes to the decreased levels of alanine (see “similarities” in Tables 2 and 3) that have been reported in both HIV infection (Peltenburg et al., 2018; Sitole et al., 2019) and TB (Zhou et al., 2013; Vrieling et al., 2018; Vrieling et al., 2019). The decrease in alanine relates to cachexia in HIV-positive and TB patients, as a study that included lung cancer patients related aberrations in glucose and alanine metabolism to the degree of weight loss, rather than the presence of cancer (Leij-Halfwerk et al., 2000). In other tissues, the carbon skeletons of amino acids (except for leucine and lysine) are also used for gluconeogenesis. Such oxidation of amino acids results in the need for increased ammonia secretion through the urea cycle (Dashty, 2013), previously mentioned to be dysfunctional (due to mitochondrial dysfunction or other mechanisms), as per the urea cycle alterations discussed above.

Histidine is associated with anti-inflammatory and anti-oxidative processes (Li et al., 2018), mainly through the actions of or effects on cytokines (Son et al., 2005; Hasegawa et al., 2012). As such, decreased levels of this essential amino acid have been associated with inflammatory diseases (Hisamatsu et al., 2012; Yu et al., 2015) including HIV infection (Li et al., 2018) and TB (Weiner et al., 2012; Vrieling et al., 2018; Vrieling et al., 2019) (see “similarities” in Table 3). The levels of histidine were found to be significantly correlated with CD4 T cell count, which implies a role for this amino acid in CD4 T cell regeneration (Li et al., 2018).

A metabolite directly associated with OS, which was common amongst HIV infection (Williams et al., 2011) and TB (Weiner et al., 2012; Che et al., 2013) metabolomics studies, was pyroglutamic acid (also referred to as 5-oxoproline) (see “similarities” in Table 2). The gamma-glutamyl cycle (γ-GC) forms an integral part of glutathione recycling. Gamma-glutamyl dipeptides, by-products of glutathione synthesis, have been reported to be lower in the serum of TB patients compared to healthy controls (Weiner et al., 2012; Vrieling et al., 2019). Similarly, 5-oxoproline, a metabolite directly downstream from the gamma-glutamyl amino acid in the γ-GC, shows the same trend (Che et al., 2013; Combrink et al., 2019) and correlates with pathological lung damage (Che et al., 2013). The imbalance of the γ-GC during active TB suggests decreased antioxidant capacity because of the prolonged OS. The alterations observed for other amino acids in HIV infection, such as glutamate and aspartate, have also been associated with OS (Sitole et al., 2019). In serum, decreased methionine and increased oxidative products of this amino acid (sulfoxymethionine) have been reported for TB (Suzuki et al., 2016; Ding et al., 2020), but not HIV infection (“differences,” Table 2). Decreased methionine has been reported for HIV infection in plasma (Peltenburg et al., 2018) but this is an inconsistent finding. Based on previous reports (using conventional techniques), OS-associated alterations are worse during the co-infected state (Madebo et al., 2003; Awodele et al., 2012). However, the mechanisms that give rise to these alterations may interact in more complex ways than that which would result in simple compounding of effects.

Immune cells remodel energetically and structurally upon immune activation to accomplish the appropriate differentiation. This results in metabolic changes associated with increased energetic and biosynthetic demands as the immune system responds to the increased viral load (Plaza et al., 2016). Mitochondrial dysfunction affects multiple systems (Nunnari and Suomalainen, 2012) contributing to the loss of lipid homeostasis, as this organelle plays key roles in the biosynthesis of phospholipids for membranes, as well as the catabolism of fatty acids (Tilokani et al., 2018). Immune cell function is thus impacted, as phospholipids are essential in the structural remodeling [especially glycerophospholipids and phosphatidylcholines (Sonnino et al., 2018)], differentiation, and cell signaling of immune cells, all of which are crucial to mounting an effective immune response (O'Donnell et al., 2018). As such, it is not surprising that alterations to phospholipids are a common finding in HIV infection and TB metabolic profiles (“similarities,” Table 2). Increased cholesterol esters and decreased phospholipids in TB patients have also been associated with the utilization of host lipids by *Mtb* as a carbon source and in various immune evasion mechanisms. Furthermore, the combination of two phosphatidylcholines, a cholesterol ester, and sphingomyelin, was able to distinguish TB patients from those with other lung conditions (Han et al., 2020).

Phosphatidylcholines (phospholipids, Table 2) have also been implicated as markers of a more progressive/worsened outcome in HIV infection and TB. In a cohort of HIV-positive individuals on ART, a decrease in phosphatidylcholines predicted poor immunological recovery (Rosado-Sanchez et al., 2019). Interestingly, these lipids were restored after 1 year of ART (Peltenburg et al., 2018). Huang et al. (2020) searched for serum metabolic biomarkers of MDR-TB, as alternative diagnostic options in future. Decreased NADH metabolites, phosphatidylcholines, caprylic acid, and increased D-xylulose in serum distinguished MDR-TB patients from drug-susceptible TB patients and healthy controls. The authors speculated that these alterations may represent mechanisms of drug resistance, especially relating to cell membrane remodeling in resistant strains of *Mtb*.

Alterations to fatty acids were common between HIV infection and TB in serum (see “similarities” in Table 2). Viral infections are associated with fatty acid synthesis (Plaza et al., 2016), which, in addition to various other factors such as mitochondrial dysfunction, contribute to a profile of increased unsaturated triglycerides and decreased sphingomyelin species, as observed by Peltenburg et al. (2018). Alterations to acylcarnitine metabolism, which is linked to fatty acid metabolism, during HIV infection are suggestive of mitochondrial dysfunction (Li et al., 2018). The importance of fatty acids as indicators of TB is exemplified by Che et al. (2018) and Cao et al. (2021). These studies showed that increased levels of tryptophan catabolic products, decreased fatty acids, as well as increased acylcarnitines and bile acids could distinguish TB pleural effusion (TPE) from pleural effusion caused by lung cancer (malignant pleural effusion; MPE), a condition which is also characterized by inflammation and altered energy metabolism. A combination of four acylcarnitines and two fatty acids discriminated TPE from MPE with the highest sensitivity and specificity (Cao et al., 2021). Fatty acids have already been proven to be important in the context of HIV/TB co-infection, as polyunsaturated fatty acids, glycerophospholipids, and sphingomyelins distinguished individuals who developed IRIS from those who did not (Silva et al., 2019).

The metabolism of choline, sarcosine and creatinine is closely linked, as all three these metabolites are intermediates in pathways related to glycine metabolism. While the plasma levels of choline were increased during HIV infection (Munshi et al., 2013), they were lower for TB patients and even more so in TB patients with DM (Vrieling et al., 2019) (see “similarities” in Table 3). Choline is a major component of phospholipids, and as such alterations in the levels of this metabolite is associated with membrane turnover (Munshi et al., 2013), which is high during immune activation. Furthermore, choline is also a precursor for acetylcholine, a neurotransmitter, and has been suggested as a biomarker for the AIDS dementia complex (Munshi et al., 2013). A gut microbe-related choline metabolite has also been related to carotid atherosclerosis progression in HIV-positive individuals (Shan et al., 2018). Decreased levels of choline have been associated with non-alcoholic fatty liver disease due to decreased intake of choline or gut microbe dysbiosis (Leermakers et al., 2015; Sharpton et al., 2019). This trend had not been reported in TB patients before the study of Vrieling et al. (2019), who advised caution in the interpretation of this finding. Sarcosine is a downstream metabolite of choline and is converted to glycine. It is an important intermediate in one-carbon metabolism, which is involved in many physiological processes, including redox defense (notably through glutathione). Increased levels of sarcosine (see “similarities” in Table 3) have been associated with folate deficiencies and muscle wasting during HIV infection (Munshi et al., 2013), but this metabolic alteration has not been interpreted in the context of TB. However, as sarcosine is a product of muscle degradation and cachexia is a characteristic feature of TB (Semba et al., 2010), there may be a similar mechanism driving the increased levels of sarcosine. Glycine and arginine are precursors for creatine. The majority of all creatine in the body is in muscle, where it serves as the primary fuel to maintain ATP availability for cellular metabolism, especially during periods of high energy demand (Kreider and Stout, 2021). As such, the observed serum creatine in HIV infection (Sitole et al., 2019) and TB (Weiner et al., 2018) relates to the prolonged period of high energy expenditure and cachexia associated with these diseases (see “similarities” in Table 2). Another contributor to this alteration may be mitochondrial dysfunction, as creatine crosses the mitochondrial membrane as part of the creatine phosphate shuttle, which facilitates the replenishment of ATP (Kreider and Stout, 2021).

**FIGURE 1:**
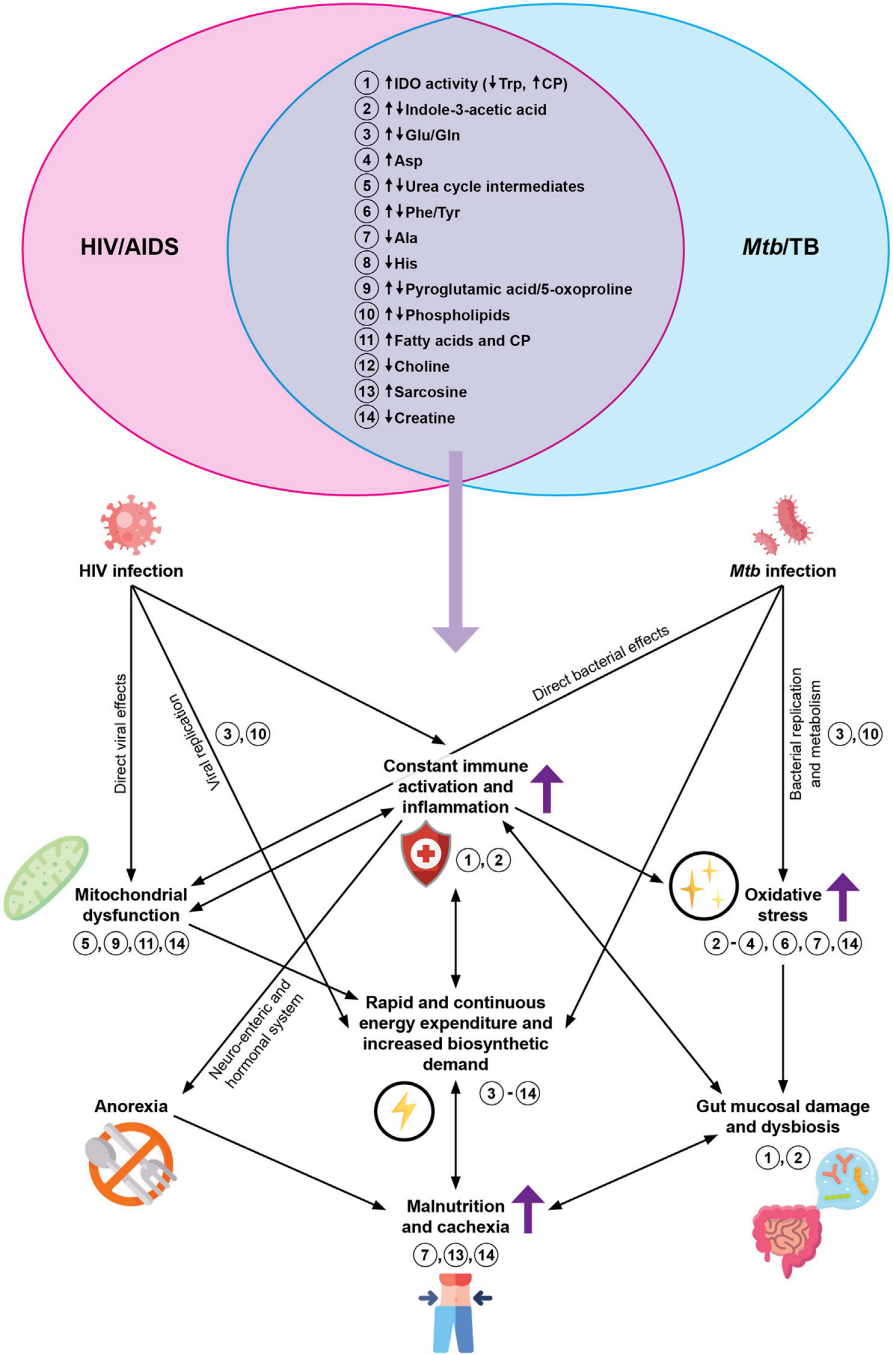
The proposed metabolic effects of HIV/TB co-infection, based on the overlapping findings of prior blood-based HIV infection and TB metabolomics studies (as indicated by the Venn diagram at the top). The dark purple arrows represent effects that are known to be exacerbated during HIV/TB co-infection. The light purple arrow represents metabolites hypothesized to be altered during HIV/TB co-infection, based on published literature. The numbers indicate the overlapping metabolites and where they play a role in what is known about host metabolism during HIV/TB co-infection. Abbreviations: HIV, human immunodeficiency virus; AIDS, acquired immunodeficiency syndrome; *Mtb*, *Mycobacterium tuberculosis*; TB, tuberculosis; IDO, indoleamine-2,3-dioxygenase; Trp, tryptophan; CP, catabolic products; Glu, glutamate; Gln, glutamine; Phe, phenylalanine; Tyr, tyrosine; Asp, aspartate; Ala, alanine; His, histidine.

Considering the comparisons (Tables 2 and 3), the HIV/TB co-infected metabolic profile obtained from blood is likely to be characterized primarily by exacerbated amino acid alterations (especially the essential amino acids and those non-essential amino acids involved in immune function and redox balance such as glutamate and glutamine). There may also be lipid alterations (deduced from similar metabolic changes highlighted in Tables 2 and 3). Figure 1 represents the overlapping metabolic alterations and maps these changes and their interpretations, which have been identified by HIV infection and TB metabolomics, separately, onto what is known about host metabolism during the co-infected state (dark purple arrows). It shows that HIV infection and TB (to the left and right of the Venn diagram, respectively) is associated with constant immune activation and inflammation as well as alterations to the gut microbiome (metabolites 1 and 2), which increases the host energy expenditure as immune cells differentiate (reflected by metabolites 3–14) and pathogens carry out biosynthetic processes (metabolites 3 and 10). This adds to the oxidative profile (metabolites 2–4, 6, 7 and 14), impacting mitochondrial structure and function (metabolites 5, 9, 11 and 14). Part of the urea cycle occurs in mitochondria, and since this organelle’s structure and function is impacted, there is reduced clearance of ammonia, which translates to urea cycle alterations. In this chronic state of immune activation and inflammation, the individual experiences malaise and anorexia, leaving a malnourished individual who eventually suffers from cachexia (metabolites 7, 13 and 14). Dysfunctional mitochondria impact lipid metabolism and is reflected as changes in phospholipids, choline, sarcosine and fatty acids—all with interrelated and sometimes compounding effects on the immune system, inflammation and biosynthetic processes. Given that these changes occur during HIV infection and TB respectively, it is highly likely that they will be detected in co-infected samples (represented by the overlapping Venn diagram) and that more quantitative versus qualitative differences will reflect.

Although the lipids did not overlap between the studies compared, the classes affected were similar, and their numerous roles in HIV infection, TB, and the immune response are already established. The exacerbated immune dysfunction during co-infection (Sharan et al., 2020) is likely to be reflected in the metabolic profile in terms of the overlapping HIV infection and TB metabolites (Figure 1 and “similarities” in Tables 2–4). Furthermore, those altered metabolites which have to date only been associated with either HIV infection or TB may also be significant during co-infection (“differences” in Tables 2–4) and metabolites that have not previously been associated with HIV infection or TB may also be significant in the co-infection metabolic profile.

Features unique to TB pathology have also been reported, such as fibrinopeptides, proposed to originate from the granuloma (Weiner et al., 2012) (see “differences,” Table 2). Within plasma, alterations to the branched-chain amino acids were more commonly reported for HIV infection than for TB, although alterations to these amino acids are commonly attributed to immune activation and wasting, which are also features of TB (see “differences,” Table 3). Given the limited number of metabolomics studies that focused on HIV/TB co-infection to date, the metabolic mechanisms underlying HIV/TB co-infection remains incompletely characterized. The comparison made here yields insight into how the metabolic changes during HIV infection and TB may interact during the co-infected state and could serve to inform future study design. Compared to blood, urine is used less frequently for characterization or diagnostic purposes of either HIV infection or TB, although it has been popular for TB drug metabolism elucidation (see Du Preez et al., 2019). As such, the extensive comparison made for blood could not be made for urine (and other sample matrices), and hence a large piece of the puzzle is still missing.

Given the advantages of urine as a clinical specimen (Du Preez et al., 2019), it has been considered an excellent alternative to sputum for TB diagnostics. Characterizing the urine metabolic profile of HIV-positive individuals will be a necessary step if urine were to be developed as a robust TB diagnostic matrix. The HIV infection and TB urine metabolic profiles overlap to a smaller degree than the blood-based profiles. While the urinary metabolic profile of HIV-positive patients indicates altered energy metabolism and the utilization of amino acids for energy, the urinary TB metabolic profile indicates increased inflammation and compromised insulin production in addition to reflecting *Mtb*-related alterations (see “differences,” Table 4). This relatively smaller degree of overlap may be due to the limited number of studies eligible for the comparison (in which participants were untreated for either condition; see Table 4). Nevertheless, increased levels of neopterin (which has been detected using conventional assays for both diseases) and catecholamines were points of similarity in the urine metabolic profiles of HIV infection and TB (Table 4).

The study of Munshi et al. (2013) provides unique insights into HIV infection since it relied on the use of various sample types, namely plasma, urine, and saliva. The only other urinary HIV-related metabolomics study is that of Chetwynd et al. (2017), which does not concur with Munshi et al. (2013) on all results. For instance, the authors’ observations regarding the extent to which immunosuppression is reflected in urine differs. Chetwynd et al. (2017) found that no separation could be achieved within the HIV-positive treatment-naïve group upon stratification by CD4 T cell count (point of division at 250 cells/mm^3^), which led them to suggest that metabolic markers of immunosuppression are not visible in urine. However, conventional methodologies established that urinary neopterin has an inverse correlation with CD4 T cell count (Immanuel et al., 2005). Increased levels of urinary neopterin have been recognized as a marker of immune activation, a trend which has been observed in urinary HIV infection (Munshi et al., 2013) and TB metabolomics studies, as well as in a small group of co-infected patients included in the study of Isa et al. (2018), in which the elevation was more pronounced. These results confirm previous findings [reviewed by Siawaya et al. (2007)] and suggest that urinary neopterin may be useful as part of the HIV/TB co-infection biosignature. There have been observations where urinary neopterin decreased in response to ART (Munshi et al., 2013) as well as in serum upon anti-TB treatment. This suggests the additional usefulness of neopterin as a prognostic marker. In co-infected patients, consistently high neopterin levels have been associated with HIV disease progression and poor outcomes (Immanuel et al., 2005). As co-infection metabolic profiles have not yet been systematically studied, this remains a viable avenue for future investigation.

There are a few studies not relevant to the comparisons in Tables 2–4, but which are important to mention in the context of the co-infected state. Alterations to the human microbiome have been associated with both HIV infection and TB, but it remains unclear exactly how the microbiome is affected during co-infection. The composition of the microbiome of the upper respiratory tract of HIV-positive treatment-naïve individuals was similar to those of HIV/TB co-infected patients except for a lower abundance of species belonging to the *Veillonellaceae* family of bacteria. This family was also less abundant in the treatment-naïve TB group relative to the treated TB group. This suggests such an alteration to the microbiome of HIV-positive patients to be indicative of TB, but this requires further investigation (Htun et al., 2018).

Two metabolomics studies focused on lung health in HIV-positive individuals, which provides the opportunity to evaluate the state of the lungs before *Mtb* infection in HIV-positive individuals from a metabolomics perspective. Cribbs et al. (2014) studied the BALF metabolic profiles of HIV-positive individuals compared to healthy controls and found that the primary differences reflected in metabolites have their origin in the lung microbiota, and could further indicate a subclinical infection in the HIV-positive lung. This study established a specific metabolic profile of the HIV-positive lung environment, which gives an indication of lung immune function and could contribute to improving diagnostic and prognostic techniques. In another study, HIV-positive individuals could not be distinguished from healthy controls based on lung microbial population composition (determined by 16S rRNA gene sequencing); however, a distinction could be made based on the BALF metabolic profile (Cribbs et al., 2016). These studies indicate that the interaction of the host and the microbiota contribute to a functional difference in the HIV-positive lung, rather than the species present. Given that HIV-induced metabolic alterations have their origin in immune dysfunction, it is the most likely cause for the alterations observed by the authors. Such an altered environment may change the way that the HIV-positive host interacts with *Mtb*, and vice versa, contributing to the altered clinical course and phenotype of co-infected individuals.

## Metabolomics as a Tool to Investigate Human Immunodeficiency Virus/Tuberculosis Co-Infection

There are several aspects in the HIV/TB co-infection field that remain unanswered. These gaps were reinforced/emphasized in our analysis of the HIV infection and TB metabolomics literature on informing potential changes during co-infection. The overlap in metabolites from the respective HIV infection and TB studies which confirm what is already known about co-infection indicates the potential use of metabolomics in further characterizing the co-infected state.

The diagnostic problems experienced in HIV/TB co-infected individuals are related to the diagnosis of TB rather than the diagnosis of HIV infection, as mentioned above, due to the limitations of sputum smear and culture-based tests [reviewed by Schutz et al. (2010); Tiberi et al. (2017)]. Co-infected individuals are also often unable to provide a sufficient sputum sample for diagnostic testing, especially when severely immunocompromised (World Health Organization, 2015). Molecular tests for TB diagnosis (GeneXpert MTB/RIF assay and the improved version, GeneXpert MTB/RIF Ultra) are highly effective in diagnosing TB in HIV-negative individuals, has a better turn-around time, and can give an early indication of drug resistance. However, access to such tests in high burden areas is limited by cost and there are concerns about the specificity (false-positives) of this test in HIV-negative individuals (World Health Organization, 2019). Similarly, a urinary point-of-care diagnostic assay based on the detection of an *Mtb* cell wall component, lipoarabinomannan (LAM), has not found widespread application partly due to its inapplicability to HIV-negative samples. It is recommended that this test not be used for screening or diagnosis in HIV-negative individuals nor HIV-positive individuals with relatively high CD4 T cell counts (World Health Organization, 2015). Given the low sensitivity of this test, an improved version (Fujifilm SILVAMP TB LAM) has been developed which retained specificity with improved diagnostic sensitivity (Broger et al., 2019). Despite high efficacy when this test is applied to immunocompromised individuals, a difficulty remains in assigning diagnostic categories [such as definite TB, possibly TB, or not TB, as used by Broger et al. (2019)] based on the results of various indicators of TB. For example, several patients could not be diagnostically classified, but tested positive using Fuji-LAM. Consequently, these patients were not started on anti-TB drugs and died within 3 months of follow-up (Broger et al., 2019).

Considering the sputum diagnostic limitations, the focus for the development of new TB diagnostic techniques should be on the identification of non-sputum biomarkers. Metabolomics can aid in identifying markers with diagnostic application or be combined with existing tests to increase sensitivity and specificity. Urine is an ideal sample in this sense as the procedures for collection and storage are simple and require minimal biosafety considerations (World Health Organization, 2015). This makes identifying markers to be used in conjunction with the LAM assay an attractive avenue of research to improve the early detection of TB and timely intervention in HIV-positive individuals. However, the criteria by which such a marker is selected must be carefully considered, particularly amenability to accurate and sensitive detection by an inexpensive point-of-care platform (Walzl et al., 2018) and specificity to TB (as opposed to a general inflammatory marker). One must also consider the increased incidence of extrapulmonary TB in this regard. Often, extrapulmonary TB is not included in the TB diagnostic testing procedure, and even if it was, sample collection for diagnosis remains difficult (Denkinger et al., 2014). If biomarkers specific to extrapulmonary disease are identified, including these in the standard TB diagnostic repertoire may significantly improve the early diagnosis of TB in HIV-positive individuals.

Metabolomics has been applied to identify metabolic determinants that indicate conversion of latent *Mtb* infection to clinical disease in the studies of Weiner et al. (2018) and Albors-Vaquer et al. (2020). The efficacy of such a profile has not yet been tested in HIV-positive individuals, for whom the risk of conversion to clinical disease is much greater than for HIV-negative individuals. Untreated profiles aid in understanding the underlying mechanisms that drive conversion of latent *Mtb* infection to active TB in HIV-positive individuals and provide early prognostic information, especially regarding TB-IRIS and treatment response. However, this might prove difficult considering sample availability, given the recommendation for early treatment of latent *Mtb* infection (World Health Organization, 2016a) as well as the “Test and Treat” policy for HIV infection, recommended by the World Health Organization (World Health Organization, 2016b). Nonetheless, such basic understanding is necessary for the development of robust and sustainable diagnostic and prognostic procedures.

Even if a highly effective diagnostic technique for TB in HIV-positive persons is developed, its success will depend on the integration of HIV infection and TB services on the ground, especially in high-burden areas, which are unfortunately also most often resource-limited. Once the difficult diagnosis is made, various factors complicating the management of HIV/TB co-infection come into play. The management of HIV/TB co-infection is challenging and is usually associated with unfavorable treatment outcomes due to various factors, reviewed by Tiberi et al. (2017) and Letang et al. (2020). Pharmacometabolomics is a branch of metabolomics wherein the effects of a drug or combination of drugs underlie the observed metabolic alterations (Combrink et al., 2019) and have been applied to HIV infection (Chetwynd et al., 2017; Li et al., 2018; Babu et al., 2019) and TB (reviewed by Du Preez and Loots. (2018), see Table 3). The interactions between ART and anti-TB drugs, and their interactions with enzymes, have been characterized by high-performance LC-MS (Wenning et al., 2009) and LC-MS/MS (Varshneya et al., 2019), demonstrating how metabolomics aids the understanding of *in vivo* drug metabolism and efficacy, which is essential to the development of better treatment strategies for co-infected patients.

Besides the interactions of the drugs themselves, variations on an individual level, identified as one of the factors contributing to TB treatment failure (De Villiers and Loots, 2013) will also affect the efficacy of drug regimens. The rate of recurrent TB (relapse and reinfection) is greater among HIV-positive compared to HIV-negative individuals (Sonnenberg et al., 2001; Charalambous et al., 2008). The only way to prevent reinfection would be an effective vaccine. Also, by better understanding the mechanisms underlying treatment failure in co-infected patients, the cases of relapse will be minimized by optimizing treatment regimens. Metabolomics has been used to identify the host factors that contribute to TB treatment failure in HIV-negative TB patients (Luies et al., 2017). Such an approach could provide valuable insights, as co-infection is associated with poor treatment outcomes and susceptibility to infection in general, due to the additional immunosuppressive effects of HIV (Guerra-Assuncao et al., 2015). Another aspect of interest from a metabolomics perspective is how the effect of the second *Mtb* co-infection event on the HIV-positive host would compare to that of the first.

Given that much of the HIV and *Mtb* infection-induced metabolic changes are driven by immune activation, inflammation and OS with subsequent impact on the mitochondria and the gut (Figure 1), metabolomics could aid in elucidating the effect that *Mtb* has on the gut mucosal damage as induced by HIV. The metabolic markers obtained are not unique and have been reported in the metabolomics investigations of other inflammatory conditions (Leermakers et al., 2015; Pett et al., 2017). As such, more longitudinal studies, especially in the treated context, with the inclusion of more disease controls to get more specific markers associated with each condition are thus needed.

## Concluding Remarks

For the rational design of novel or improved diagnostic, treatment, and monitoring techniques, the research must first focus on better characterizing a disease state so that the origin of identified markers is understood, as opposed to unqualifiedly advocating statistically significant markers that have no true specificity for the disease state. It is of extreme importance to develop and validate highly specific biomarkers for HIV/TB co-infection—although biosignatures may be more practical considering the overlapping pathologies of HIV infection and TB. Furthermore, when developing these metabolic signatures, it should be kept in mind that the ultimate goal is point-of-care, individualized assessments that should be accessible even in resource-limited settings, as these are commonly the areas of high incidence and spread. This further requires the complete integration of HIV infection and TB services and for this, a greater awareness of co-infection should be brought among clinicians and built into infrastructure to ensure that even those with minimal education could apply it.
